# Complete chloroplast genome sequence of *Bambusa subtruncata* (Bambusodae)

**DOI:** 10.1080/23802359.2020.1781577

**Published:** 2020-06-26

**Authors:** Yangyang Zhang, Lili Fan, Dejin Xie, Muhammad Waqqas Khan Tarin, Jundong Rong, Tianyou He, Yushan Zheng

**Affiliations:** a College of Forestry, Fujian Agriculture and Forestry University, Fuzhou, PR China; b College of Arts & College of Landscape Architecture, Fujian Agriculture and Forestry University, Fuzhou, Fujian, PR China

**Keywords:** *Bambusa subtruncata*, plastid genome, phylogeny, Bambusodae

## Abstract

*Bambusa subtruncata* is found in Xinyi county, Maoming city, Guangdong province, China. In the current study, we sequenced the complete chloroplast genome of *B. subtruncata* and reported for the first time. The genome was 139,444 bp in total length, including a large single-copy (LSC) region of 82,956 bp, a small single-copy (SSC) region of 12,897 bp, and a pair of invert repeats (IR) regions of 21,798 bp. Plastid genome comprised of 127 genes in total; 82 protein-coding genes, 37 tRNA genes, and eight rRNA genes. Phylogenetic analysis based on 25 chloroplast genomes indicates that *B. subtruncata* is closely related to *Bambusa emeiensis* in Bambusodae.

*Bambusa subtruncata* is one of the excellent ornamental bamboo species with good cold resistance. The bamboo leaves become yellow and whorl under cold conditions, but this species recovers quickly without impacting the growth and landscape (Teng 2013). *B. subtruncata* can normally grow 4–5 meters tall with a diameter of 2–2.5 cm, and an internode length of 25–30 cm. It is very similar to Huamei Bamboo in appearance. The chloroplasts (cp) genome has a maternal inheritance and conserved structure, which has been used for investigating evolutionary and phylogenetic relationships of plants (Wang et al. 2018). Therefore, we reported the complete chloroplast genome (cp) of *B. subtruncata* based on Illumina pair-end sequencing data. Fresh leaves tissues of *B. subtruncata* were collected from Fujian province, China (Fujian Agriculture and Forestry University, Bamboo Garden, Fuzhou: 119°14´16′´E, 26°5´7′´N) and dried into silica gel instantaneously. The specimens were kept in the Herbarium of College of Forestry, Fujian Agriculture and Forestry University (specimen code 101301). DNA was extracted from fresh leaves tissues, with 500 bp randomly interrupted sequence by the Covaris ultrasonic breaker for library construction. The constructed library was sequenced PE150 by Illumina Hiseq Xten platform, with ∼2GB data generated. Illumina data were filtered by a script in the cluster (default parameter: -L5, -p0.5, -N0.1). The complete plastid genome of *Arundinaria faberi* (GeneBank accession: JX513414) as reference and plastid genome of *B. subtruncata* was assembled by GetOrganelle pipe-line (https://github.com/Kinggerm/GetOrganelle). It can get the plastid-like reads, and the reads were viewed and edited by Bandage (Wick et al. 2015). The cp genome annotation was assembled based on the comparison by Geneious v 11.1.5 (Biomatters Ltd, Auckland, New Zealand) (Kearse et al. 2012). The annotation result was drawn with the online tool OGDRAW (http://ogdraw.mpimp-golm.mpg.de/) (Lohse et al. 2013).

The complete plastid genome sequence of *B. subtruncata* (GenBank accession: MT517441) was 139,444 bp in length, with a large single-copy (LSC) region of 82,956 bp, a small single-copy (SSC) region of 12,897 bp, and a pair of inverted repeats (IR) regions of 21,798 bp. The complete chloroplastid genome contained 127 genes, including 82 protein-coding genes, 37 tRNA genes, and eight rRNA genes. The complete genome GC content was 38.9%. To reveal the phylogenetic position of *B. subtruncata* with other members of Bambusodae, we performed a phylogenetic analysis based on 23 complete chloroplast genomes of Bambusodae, and two taxa (*Arundinaria fargesii, Arundinaria gigantea*) as outgroups. All of them were downloaded from NCBI GenBank. The sequences were aligned by MAFFT v7.307 (Katoh and Standley 2013), and the phylogenetic tree was constructed by RAxML (Stamatakis 2014). The phylogenetic tree revealed that *B. subtruncata* was most closely related to *Bambusa emeiensis* with strong support (Figure 1).

**Figure 1.**
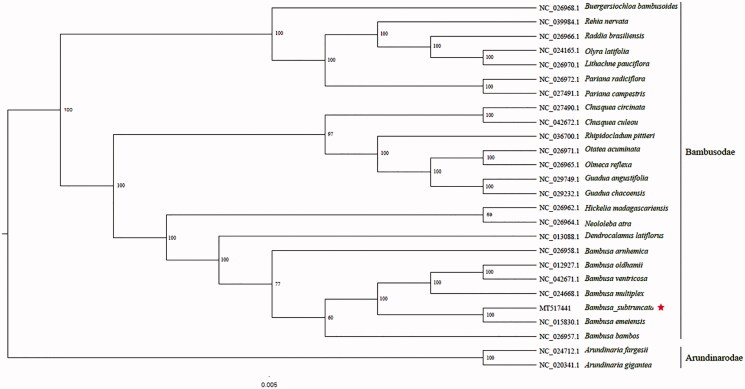
Phylogenetic analysis of 23 species of Bambusodae and two taxa (*Arundinaria fargesii, Arundinaria gigantea*) as outgroup based on plastid genome sequences by RAxML, bootstrap support value near the branch.

## Data Availability

The data that support the findings of this study are openly available at https://www.ncbi.nlm.nih.gov/ GeneBank with following accession number MT 517441.
